# Nuclear Hormone Receptor Activity of Polybrominated Diphenyl Ethers and Their Hydroxylated and Methoxylated Metabolites in Transactivation Assays Using Chinese Hamster Ovary Cells

**DOI:** 10.1289/ehp.0900753

**Published:** 2009-04-28

**Authors:** Hiroyuki Kojima, Shinji Takeuchi, Naoto Uramaru, Kazumi Sugihara, Takahiko Yoshida, Shigeyuki Kitamura

**Affiliations:** 1 Hokkaido Institute of Public Health, Sapporo, Japan; 2 Graduate School of Biomedical Sciences, Hiroshima University, Hiroshima, Japan; 3 Nihon Pharmaceutical University, Saitama, Japan; 4 Asahikawa Medical School, Asahikawa, Japan

**Keywords:** androgen receptor, brominated diphenyl ether, Chinese hamster ovary cells, estrogen receptor, glucocorticoid receptor, reporter gene assay, thyroid hormone receptor

## Abstract

**Background:**

An increasing number of studies are reporting the existence of polybrominated diphenyl ethers (PBDEs) and their hydroxylated (HO) and methoxylated (MeO) metabolites in the environment and in tissues from wildlife and humans.

**Objective:**

Our aim was to characterize and compare the agonistic and antagonistic activities of principle PBDE congeners and their HO and MeO metabolites against human nuclear hormone receptors.

**Methods:**

We tested the hormone receptor activities of estrogen receptor α (ERα), ERβ, androgen receptor (AR), glucocorticoid receptor (GR), thyroid hormone receptor α_1_ (TRα_1_), and TRβ_1_ against PBDE congeners BDEs 15, 28, 47, 85, 99, 100, 153, and 209, four *para*-HO-PBDEs, and four *para*-MeO-PBDEs by highly sensitive reporter gene assays using Chinese hamster ovary cells.

**Results:**

Of the 16 compounds tested, 6 and 2 showed agonistic activities in the ERα and ERβ assays, respectively, and 6 and 6 showed antagonistic activities in these assays. 4′-HO-BDE-17 showed the most potent estrogenic activity via ERα/β, and 4′-HO-BDE-49 showed the most potent anti estrogenic activity via ERα/β. In the AR assay, 13 compounds showed antagonistic activity, with 4′-HO-BDE-17 in particular inhibiting AR-mediated transcriptional activity at low concentrations in the order of 10^−8^ M. In the GR assay, seven compounds, including two HO-PBDEs and two MeO-PBDEs, showed weak antagonistic activity. In the TRα_1_ and TRβ_1_ assays, only 4-HO-BDE-90 showed weak antagonistic activity.

**Conclusions:**

Taken together, these results suggest that PBDEs and their metabolites might have multiple endocrine-disrupting effects via nuclear hormone receptors, and *para*-HO-PBDEs, in particular, possess more potent receptor activities compared with those of the parent PBDEs and corresponding *para*-MeO-PBDEs.

Polybrominated diphenyl ethers (PBDEs) are used in large quantities worldwide as a flame retardant additive in plastic materials, paints, textile fabrics, and so forth (Alaee et al. 2003; Darnerud et al. 2001; de Wit 2002; Sjödin et al. 2003). Because of their leachability from various materials and their persistence, PBDEs have been identified in various environmental sectors, including sediment and fish from rivers (Sellström et al. 1998), indoor air (Saito et al. 2007; Wilford et al. 2004), and house dust (Stapleton et al. 2005). Moreover, PBDEs have been identified in human tissue samples, such as blood and breast milk, because of their bioaccumulative nature (Mazdai et al. 2003; Schecter et al. 2003). Recently, great concern has arisen about the possible impact on health of PBDE exposure because studies have revealed rising PBDE levels in the tissue from humans and wildlife (Hites 2004; Law et al. 2003). Reports have indicated that the PBDE congeners most commonly found in human samples are 2,2′,4,4′-tetra bromodiphenyl ether (BDE-47), 2,2′,4,4′,5-pentabromodiphenyl ether (BDE-99), 2,2′,4,4′,6-pentabromodiphenyl ether (BDE-100), and 2,2′,4,4′,5,5′-hexa bromodiphenyl ether (BDE-153), with BDE-47, in particular, being one of the predominant congeners found in humans (Hites 2004; Mazdai et al. 2003). In addition, more recent studies have shown that hydroxylated PBDEs (HO-PBDEs) and methoxylated PBDEs (MeO-PBDEs) are formed as metabolites from parent PBDE congeners by oxidizing enzymes, such as cytochrome P450 (CYP), and are also present in the plasma of wild animals (Verreault et al. 2005) and humans (Athanasiadou et al. 2008; Qui et al. 2009) and in human breast milk (Lacorte and Ikonomou 2009).

Nuclear hormone receptors are ligand-dependent transcription factors that regulate a variety of important physiologic processes (McKenna et al. 1999). Several studies have reported that nuclear hormone receptors such as estrogen receptor (ER) and androgen receptor (AR) interact with xenobiotic chemicals, for example, persistent pesticides and polychlorinated biphenyls (PCBs) (Bonefeld-Jørgensen et al. 2001; Kelce et al. 1995; Kojima et al. 2003; Sohoni and Sumpter 1998; Vinggaard et al. 1999). This implies that these chemicals may disrupt the endocrine system via a variety of nuclear hormone receptors, and PBDEs may display toxicity similar to that of PCBs because of their structural resemblance. To date, *in vitro* and *in vivo* studies have reported that several PBDE congeners possess estrogenic and antiestrogenic, and/or antiandrogenic activity (Hamers et al. 2006; Meerts et al. 2001; Stoker et al. 2005). However, although estrogenic responses are mediated via two subtypes of ERα and ERβ, which differ in their ligand binding ability and transactivation properties (Kuiper et al. 1997; McInerney et al. 1998), the estrogenicity of PBDEs has been evaluated on the basis of their effects via ERα, but not via ERβ. In addition, little work has been done on the agonistic and antagonistic activity of PBDEs against glucocorticoid receptor (GR) and thyroid hormone receptors (TRs) other than ERs and AR, and the nuclear hormone receptor activity of HO- and MeO-PBDEs is not fully understood. Therefore, further study regarding endocrine-disrupting effects of PBDEs, including HO and MeO-PBDEs, is required for assessing potential health risks.

Transactivation assays such as reporter gene assays have an advantage in detecting agonistic and antagonistic activity of various chemicals against nuclear hormone receptors. Using Chinese hamster ovary (CHO-K1) cells, we previously developed novel reporter gene assays that were highly sensitive and specific to chemicals and provided evidence that a variety of pesticides and plastisizers, such as phthalates, have both agonistic and antagonistic activities against ERα, ERβ, and AR (Kojima et al. 2003, 2004; Takeuchi et al. 2005). Our recent study (Takeuchi et al. 2009) provided comparative data on GR and TRα_1_/β_1_ activity in addition to ERα/β and AR activities for various phyto chemicals in same the assay systems using CHO-K1 cells. In the present study, to elucidate the endocrine-disrupting property of PBDEs and their HO and MeO metabolites, we characterized the agonistic and antagonistic activity of principle PBDE congeners together with *para*-HO- and *para*-MeO-PBDEs against six hormone nuclear receptors: ERα/β, AR, GR, and TRα_1_/β_1_. We used BDEs 15, 28, 47, 85, 99, 100, 153, and 209 as principle PBDEs, and four *para*-HO-PBDEs identified in human blood (Athanasiadou et al. 2008) and/or in serum from rats and mice administered PBDE congeners such as BDEs 47, 99, 100, and 209 (Malmberg et al. 2005; Qui et al. 2007). The four *para*-MeO-PBDEs used in this study were selected because of their structural correspondence to the four *para*-HO-PBDEs, because these compounds are formed through methylation of HO-PBDEs, probably by a catechol-*O*-methyltransferase. Consequently, we found that several of the 16 tested PBDEs and metabolites have ERα and ERβ agonistic activity, and ERα, ERβ, AR, GR, TRα_1_, and TRβ_1_ antagonistic activity. In this article, we provide the first evidence that parent PBDE congeners and *para*-HO- and *para*-MeO-PBDEs have multiple effects on transcriptional activity via nuclear hormone receptors, and that they have the potential to affect both the endocrine system and reproduction in whole organisms.

## Materials and Methods

### Chemicals, biochemicals, and cells

We purchased 17β-estradiol (E_2_; > 97% pure), 5α-dihydrotestosterone (DHT; 95% pure), hydrocortisone (HC; > 98% pure), tamoxifen citrate (TAM, 98% pure), and hydroxyflutamide (HF; > 99% pure) from Wako Pure Chemical Industries, Ltd. (Osaka, Japan). 3,3′,5-Triiodo-L-thyronine (T_3_; 99% pure), tetrabrominated bisphenol A (TBBPA; 99% pure), and mifepristone (RU-486; 98% pure) were purchased from Sigma-Aldrich (St. Louis, MO, USA).

**Figure 1:**
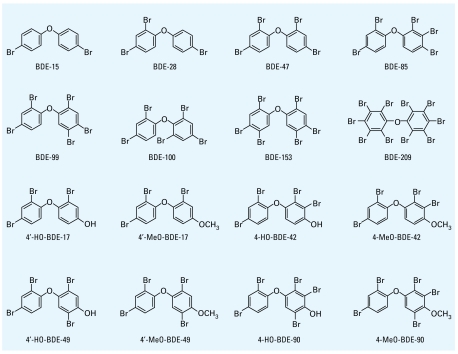
Chemical structures of the PBDEs and their HO and MeO metabolites used in the present study.

The structures of the PBDE congeners and *para*-HO- and *para*-MeO-PBDEs tested in this study are shown in Figure 1. The PBDE congeners 4,4′-dibromodiphenyl ether (BDE-15), 2,4,4′-tribromodiphenyl ether (BDE-28), BDE-100, and BDE-153 were purchased from AccuStandard, Inc. (New Haven, CT, USA). We obtained decabromodiphenyl ether (BDE-209) from Wako. We synthesized BDE-47 according to the method of Teclechiel et al. (2009), and 2,2′,3,4,4′-pentabromodiphenyl ether (BDE-85) and BDE-99 following the method of Orn et al. (1996). The PBDE metabolites 4′-hydroxy-2,2′,4-tribromo diphenyl ether (4′-HO-BDE-17), 4′-methoxy-2,2′,4-tribromodiphenyl ether (4′-MeO-BDE-17), 4-hydroxy-2,2′,3,4′-tetrabromodiphenyl ether (4-HO-BDE-42), 4-methoxy-2,2′,3,4′-tetrabromo diphenyl ether (4-MeO-BDE-42), 4′-hydroxy-2,2′,4,5′-tetrabromodiphenyl ether (4′-HO-BDE-49), 4′-methoxy-2,2′,4,5′-tetrabromodiphenyl ether (4′-MeO-BDE-49), 4-hydroxy-2,2′,3,4′,5-pentabromodiphenyl ether (4-HO-BDE-90), and 4-methoxy-2,2′,3,4′,5-pentabromodiphenyl ether (4-MeO-BDE-90) were synthesized according to the method of Marsh et al. (2003). The purities of these synthesized compounds were > 98%. We used DMSO (Wako) as a vehicle, and all compounds were dissolved in DMSO at a concentration of 10^−2^M.

We obtained Dulbecco’s modified Eagle medium (DMEM) plus Ham’s F-12 nutrient mixture and a penicillin-streptomycin solution (antibiotics) from GIBCO-BRL (Rockville, MD, USA); Chinese hamster ovary (CHO-K1) cells from Dainippon Pharmaceutical Co. Ltd. (Osaka, Japan); fetal bovine serum (FBS) and charcoal-dextran–treated FBS (CD-FBS) from Hyclone (Logan, UT, USA); and bovine serum albumin and 4-methylumbelliferyl-β-D-galactoside from Sigma-Aldrich. We maintained CHO-K1 cells in DMEM/F-12 supplemented with 5% FBS and antibiotics. All compounds were diluted to the desired concentration in an appropriate medium immediately before use. The final solvent concentration in the culture medium did not exceed 0.1%, and this concentration did not affect cell yields.

### Plasmids

We prepared the expression plasmids pcDNAERα, pcDNAERβ, pZeoSV2AR, pSG5-GR, pZeo-TRα_1_, and pZeo-TRβ_1_ encoding full-length receptor proteins and the reporter plasmids pGL3-tkERE, pIND-ARE, pGRE-tk-Luc, and pIND-TREpal as previously described (Kitamura et al. 2005b; Kojima et al. 2003, 2004; Yamamoto et al. 2000). We purchased the internal control plasmid, pCMVβ-Gal, from Clontech (Palo Alto, CA, USA).

### Transfection of plasmids to cells and luciferase activity assay

We plated the host CHO-K1 cells in 96-well microtiter plates (Nalge, Nunc, Denmark) at a density of 8,400 cells per well in phenol red-free DMEM/F-12 containing 5% CD-FBS (complete medium) 1 day before transfection. For detection of human ERα (hERα) or hERβ activity, cells were transfected with 0.63 ng pcDNAERα or with 0.63 ng pcDNAERβ, 50 ng pGL3-tkERE, and 5 ng pCMVβ-Gal per well using FuGENE 6 Transfection Reagent (Roche Diagnostics Corp., Indianapolis, IN, USA). For detection of hAR activity, cells were transfected with 2.5 ng pZeoSV2AR, 50 ng pIND-ARE, and 5 ng pCMVβ-Gal per well using FuGENE 6 Transfection Reagent. For detection of hGR activity, we transfected cells with 1.25 ng pSG5-GR, 50 ng pGRE-tk-Luc, and 5 ng pCMVβ-Gal per well using FuGENE 6 Transfection Reagent. For detection of hTRα_1_ or hTRβ_1_ activity, cells were transfected with 1.25 ng pZeo-TRα_1_ or with 1.25 ng pZeo-TRβ_1_, 50 ng pIND-TRE-pal, and 5 ng pCMVβ-Gal per well using FuGENE 6 Transfection Reagent. After a 3-hr transfection period, cells were dosed with various concentrations of the test compounds or with 0.1% DMSO (vehicle control) in complete medium. To avoid any cytotoxic effects associated with the test compounds, we performed assays for test compounds at concentrations of < 1 × 10^−5^ M. For the measurement of the antagonistic activities via hERα, hERβ, hAR, hGR, hTRα_1_, and hTRβ_1_, the test compound was added to the cell cultures together with endogenous hormones of 1 × 10^−11^ M E_2_, 1 × 10–10 M E_2_, 1 × 10–10 M DHT, 3 × 10^−8^ M HC, 1 × 10^−8^ M T_3_, and 1 × 10–8 M T_3_, respectively. After an incubation period of 24 hr, cells were rinsed with phosphate-buffered saline (pH 7.4) and lysed with passive lysis buffer (50 μL/well; Promega, Madison, WI, USA).

We measured firefly luciferase activity with a MiniLumat LB 9506 luminometer (Berthold, Wildbad, Germany) in one reaction tube with a 5-μL aliquot of the cell lysate using the Luciferase Assay System (Promega) according to the manufacturer’s instructions. The luciferase activity was normalized against the β-galactosidase activity for each treatment. Results are expressed as mean ± SD from at least three independent experiments performed in triplicate.

### β-Galactosidase activity assay

We measured β-galactosidase activity by a fluorescence method as described previously (Takeuchi et al. 2005, 2006).

### Evaluation of agonistic and antagonistic activities

To estimate the potency of the receptor agonistic activity of the tested compounds, we represented the luminescence intensity of the assay in a dose–response curve. We obtained the concentration of the compound equal to 20% of the maximal response of E_2_, DHT, HC, or T_3_ from the dose–response curve of the luminescence intensity and expressed it as 20% relative effective concentration (REC_20_). The results for the receptor antagonistic activities of the compound are expressed as 20% relative inhibitory concentration (RIC_20_); that is, the concentration of the test compounds showing 20% inhibition of the activities induced by endogenous hormones. When the agonistic or antagonistic activity of the test compound was higher than the REC_20_ or RIC_20_ value within the concentration tested (~ 1 × 10^−5^ M), we judged the PBDEs and their metabolites to be positive for agonistic or antagonistic activity against that nuclear hormone receptor.

### Statistical analysis

We used an analysis of variance (ANOVA) followed by Bonferroni correction to evaluate the differences in transcriptional levels between the control group and each of the chemical groups in the ERα, ERβ, GR, and TRα/β antagonist assays. The level of significance was *p* < 0.05. Data are presented as mean ± SD of three triplicate experiments.

## Results

### Agonistic and antagonistic activities of the PBDEs and their metabolites via ERα

**Figure 2:**
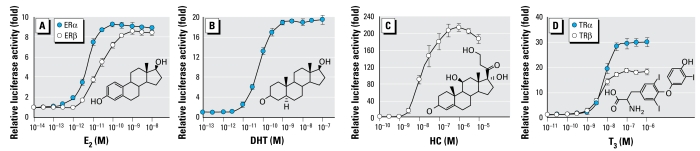
Dose–response curves for E_2_ (*A*), DHT (*B*), HC (*C*), and T_3_ (*D*) obtained from the ERα/β, AR, GR, and TRα_1_/β_1_ transactivation assays, respectively, of CHO cells transiently transfected with an expression plasmid for hERα/β, hAR, hGR, and hTRα_1_/β_1_ as well as a reporter-responsive firefly luciferase plasmid and a constitutively active β-galactosidase expression plasmid. See “Methods and Methods” for details. Values represent the mean ± SD of three independent experiments and are presented as the mean *n*-fold induction over the vehicle control.

**Figure 3:**
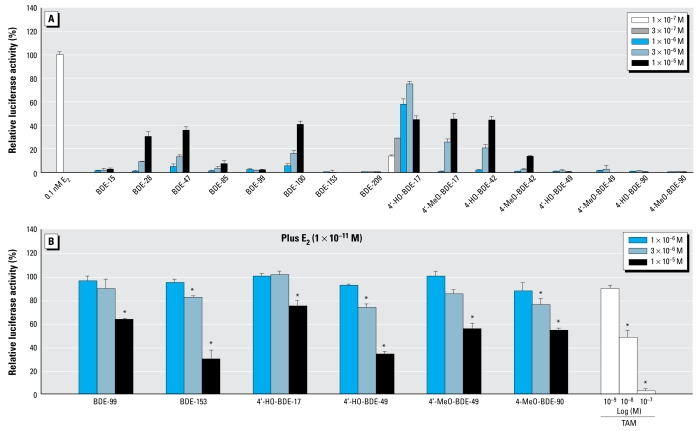
Estrogenic and antiestrogenic effects of 16 PBDEs and metabolites in the hERα transactivation assay using CHO cells transiently transfected with an expression plasmid for hERα as well as a reporter-responsive firefly luciferase plasmid and a constitutively active β-galactosidase expression plasmid. Firefly luciferase activity was normalized based on the β-galactosidase activity. (*A*) Cells were treated with 1 × 10^−7^ to 1 × 10^−5^ M of the eight PBDEs and their eight metabolites. Values represent means ± SD of three independent experiments and are presented as the percentage of the response compared with 100% activity defined as the activity achieved with 1 × 10^−10^ M E_2_. (*B*) Cells were treated with 1 × 10^−6^ to 1 × 10^−5^ M of the six compounds showing ERα antagonistic activity, or with 1 × 10^−9^ to 1 × 10^−7^ M TAM in the presence of 1 × 10^−11^ M E_2_. Values represent mean ± SD of three independent experiments and are presented as the percentage of the response, taking the activity achieved with 1 × 10^−11^ M E_2_ as 100%. *p < 0.05 (ANOVA) compared with 1 × 10^−11^ M E_2_ alone.

Figure 2A shows the dose–response curve of E_2_ from the ERα assay. From the dose–response curve, we estimated the REC_20_ value of E_2_ for ERα to be 2.5 × 10^−12^ M. As shown in Figure 3A, we found that 6 of the 16 compounds tested induced estrogenic activity greater than the 20% of the maximum activity of E_2_ in the ERα assay. The REC_20_ values of the compounds with ERα agonistic activity are described in Table 1. The relative potencies of their ERα agonistic activities descended in the following order: 4′-HO-BDE-17 ≫ 4′-MeO-BDE-17, 4-HO-BDE-42 > BDE-100 > BDE-47 > BDE-28. The estrogenic activity via ERα of 4′-HO-BDE-17 was about 100,000-fold lower than that of E_2_.

In addition, we found that 6 of the 16 compounds tested (BDE-99, BDE-153, 4′-HO-BDE-17, 4′-HO-BDE-49, 4′-MeO-BDE-49, and 4-MeO-BDE-90) had an inhibitory effect on the estrogenic activity induced by 1 × 10^−11^ M of E_2_ in the ERα assay. Figure 3B shows the dose response of these compounds and TAM, a known ER antagonist, on ERα-mediated transcriptional activity induced by E_2_. The order of relative potencies for ERα antagonistic activity was 4′-HO-BDE-49, 4-MeO-BDE-90 > BDE-153 > 4′-MeO-BDE-49 > BDE-99 > 4′-HO-BDE-17. From comparisons of their RIC_20_ values, we estimated their antiestrogenic activity via ERα to be approximately 500-fold lower than that of TAM (Table 1). These results also indicate that 4′-HO-BDE-17 possesses both ERα agonistic and antagonistic activities.

### Agonistic and antagonistic activities of the PBDEs and their metabolites via ERβ

**Figure 4:**
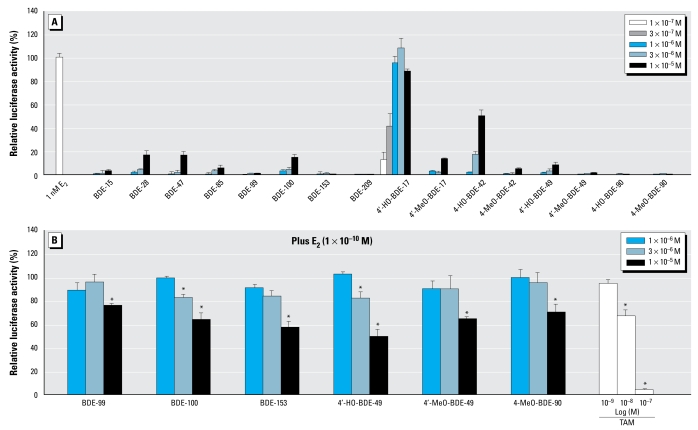
Estrogenic and antiestrogenic effects of 16 PBDEs and their metabolites in the hERβ transactivation assay using CHO cells transiently transfected with an expression plasmid for hERβ as well as a reporter-responsive firefly luciferase plasmid and a constitutively active β-galactosidase expression plasmid. The firefly luciferase activity was normalized based on the β-galactosidase activity. (*A*) Cells were treated with 1 × 10^−7^ to 1 × 10^−5^ M of the eight PBDEs and their eight metabolites. Values represent mean ± SD of three independent experiments and are presented as the percentage of the response, compared with 100% activity defined as the activity achieved with 1 × 10^−9^ M E_2_. (*B*) Cells were treated with 1 × 10^−6^ to 1 × 10^−5^ M of the six compounds showing ERβ antagonistic activity, or with 1 × 10^−9^ to 1 × 10^−7^ M TAM in the presence of 1 × 10^−10^ M E_2_. Values mean ± SD of three independent experiments and are presented as the percentage of the response, taking the activity achieved with 1 × 10^−10^ M E_2_ as 100%. *p < 0.05 (ANOVA) compared with 1 × 10^−10^ M E_2_ alone.

Figure 2A shows the dose–response curve of E_2_ from the ERβ assay. From the dose–response curve, we estimated the REC_20_ value of E_2_ for ERβ to be 5.3 × 10^−12^ M. As shown in Figure 4A, we found that of the 16 compounds tested, only 4′-HO-BDE-17 and 4-HO-BDE-42 induced estrogenic activity greater the 20% of the maximum activity of E_2_ in the ERβ assay. The REC_20_ values of 4′-HO-BDE-17 and 4-HO-BDE-42 were 2.1 × 10^−7^ M and 3.6 × 10^−^ M (Table 1), and their estrogenic activity via ERβ was about 40,000- and 680,000-fold lower than that of E_2_, respectively.

In addition, we found that 6 of the 16 compounds tested had an inhibitory effect on the estrogenic activity induced by 1 × 10^−10^ M of E_2_ in the ERβ assay. Figure 4B shows the dose responses of these six compounds and TAM on ERβ-mediated transcriptional activity induced by E_2_. The order of relative potencies for ERβ antagonistic activity was 4′-HO-BDE-49 > BDE-100, BDE-153 > 4′-MeO-BDE-49 > 4-MeO-BDE-90 > BDE-99. From comparisons of RIC_20_, we estimated their antiestrogenic activity via ERβ to be between about 500- and 1,200-fold lower than that of TAM (Table 1).

### Agonistic and antagonistic activities of the PBDEs and their metabolites via AR

**Figure 5:**
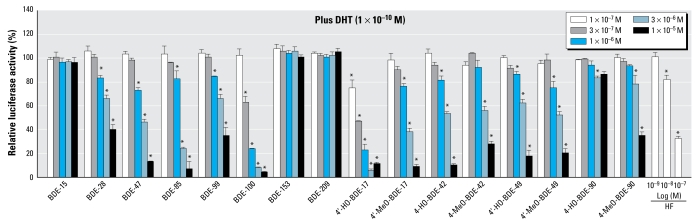
Antiandrogenic effects of 16 PBDEs and metabolites in the hAR transactivation assays using CHO cells transiently transfected with an expression plasmid for hAR as well as a reporter-responsive firefly luciferase plasmid and a constitutively active β-galactosidase expression plasmid. Cells were treated with 1 × 10^−7^ M to 1 × 10^−5^ M of 16 PBDEs and metabolites, or with 1 × 10^−9^ M to 1 × 10^−7^ M of HF in the presence of 1 × 10^−10^ M DHT to detect AR antagonistic activity. The firefly luciferase activity was normalized based on the β-galactosidase activity. Values represent mean ± SD of three independent experiments and are presented as the percentage of the response, taking the activity achieved with 1 × 10^−10^ M DHT as 100%. *p < 0.05 (ANOVA) compared with 1 × 10^−10^ M DHT alone.

Figure 2B shows the dose–response curve for DHT obtained from the AR assay. Although we examined the androgenicity of the 16 compounds in this assay, none of the compounds tested showed any AR agonistic activity (data not shown). However, we found that 12 of the 16 compounds inhibited the agonistic activity induced by 1 × 10^−10^ M DHT. Figure 5 shows the dose responses of the antagonistic activity via hAR for the 16 compounds and HF, a known AR antagonist. The order of relative potencies for AR antagonistic activity was 4′-HO-BDE-17 > BDE-100 > 4-HO-BDE-42, BDE-47 > BDE-85 > 4′-HO-BDE-49 > BDE-28 > 4′-MeO-BDE-17, BDE-99 > 4′-MeO-BDE-49, 4-MeO-BDE-42, 4-MeO-BDE-90. From a comparison of RIC_20_ values, the anti-androgenic activities of 4′-HO-BDE-17 and BDE-100 were about 5- and 10-fold lower than that of HF (1.8 × 10^−8^ M), respectively (Table 1).

### Agonistic and antagonistic activities of the PBDEs and their metabolites via GR

**Figure 6:**
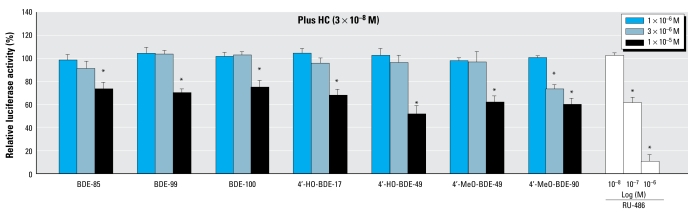
GR antagonistic effects of seven PBDEs and metabolites in the hGR transactivation assays using CHO cells transiently transfected with an expression plasmid for hGR as well as a reporter-responsive firefly luciferase plasmid and a constitutively active β-galactosidase expression plasmid. Cells were treated with 1 × 10^−7^ M to 1 × 10^−5^ M of the seven compounds showing GR antagonistic activity, or with 1 × 10^−8^ M to 1 × 10^−6^ M of RU-486 in the presence of 3 × 10^−8^ M HC to detect GR antagonistic activity. We normalized the firefly luciferase activity based on the β-galactosidase activity. Values are percentages of the response, taking the activity achieved with 3 × 10^−8^ M HC as 100%, representing means ± SD of three independent experiments. *p < 0.05 (ANOVA) compared with 3 × 10^−8^ M HC alone.

Figure 2C shows the dose–response curves for HC obtained from the GR assay. In the GR assay, although none of 16 compounds tested showed any GR agonistic activity, we found that 7 compounds, including three PBDE congeners (BDEs 85, 99, and 100), had weak GR antagonistic activity (Figure 6). The order of relative potencies for GR antagonistic activity was 4-MeO-BDE-90 > 4′-HO-BDE-49 > 4′-MeO-BDE-49 > 4′-HO-BDE-17, BDE-85 > BDE-99 > BDE-100 (Table 1). From a comparison of RIC_20_ values, we estimated all the antagonistic activities of these compounds to be > 1,000-fold lower than that of RU-486, a known GR antagonist (RIC_20_ =5.7 × 10 ^−9^M).

### Agonistic and antagonistic activities of the PBDEs and their metabolites via TRα_1_ and TRβ_1_

**Figure 7:**
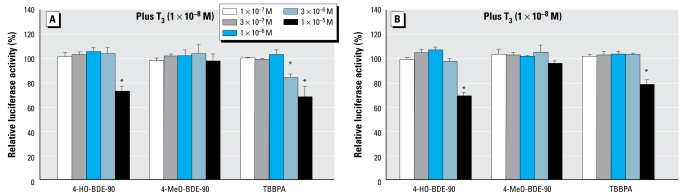
TRα_1_ (*A*) and TRβ_1_ (*B*) antagonistic effects of 4-HO-BDE-90 and 4-MeO-BDE-90 in the hTR transactivation assays using CHO cells transiently transfected with an expression plasmid for hTRα_1_ or hTRβ_1_ as well as a reporter-responsive firefly luciferase plasmid and a constitutively active β-galactosidase expression plasmid. Cells were treated with 1 × 10^−7^ M to 1 × 10^−5^ M of 4-HO-BDE-90, 4-MeO-BDE-90 or TBBPA in the presence of 1 × 10^−8^ M T_3_ to detect TR antagonistic activity. Firefly luciferase activity was normalized based on the β-galactosidase activity. Values represent mean ± SD of three independent experiments and are presented as the percentage of the response, taking the activity achieved with 1 × 10^−8^ M T_3_ as 100%. *p < 0.05 (ANOVA) compared with 1 × 10^−8^ M T_3_ alone.

Figure 2D shows the dose–response curves for T_3_ obtained from the TRα_1_/β_1_ assays. In these assays, although none of 16 compounds tested showed any TRα_1_/β_1_ agonistic activity, we found that a higher concentration (1 × 10^−5^ M) of 4-HO-BDE-90 significantly inhibited both TRα_1_- and TRβ_1_-mediated transcriptional activity induced by T_3_, whereas its corresponding metabolite 4-MeO-BDE-90 did not (Figure 7). From comparisons of RIC_20_ values for TRα_1_ and TRβ_1_ (Table 1), we estimated the antithyroid hormone activities of 4-HO-BDE-90 to be similar to those of TBBPA, previously reported to be a TR antagonist by Kitamura et al. (2005b).

## Discussion

In this study, we characterized the potential hormone receptor activity of 16 PBDEs and their HO and MeO metabolites on the basis of highly sensitive reporter gene assays using CHO-K1 cells, which we transiently transfected with expression vectors for hERα, hERβ, hAR, hGR, hTRα_1_, and hTRβ_1_, along with the appropriate reporter plasmids. The results summarized in Table 1 reveal that these compounds, excluding BDE-15 and BDE-209, exhibited not only ERα/β agonistic and AR antagonistic activities but also antagonistic activities via ERα/β, GR, and TRα_1_/β_1_.

Regarding estrogenicity via ERα of the various PBDE congeners, two studies were based on estrogen response element (ERE)-luciferase assay using a human T47D breast cancer cell line (ER-CALUX; Legler et al. 1999) for estrogenic activity testing. Meerts et al. (2001) showed that BDE-28 and BDE-100 from among the 17 PBDE congeners tested were estrogenic, and Hamers et al. (2006) reported that estrogenic responses were found for lower brominated diphenyl ethers such as BDEs 28, 47, and 100, among 19 PBDE congeners tested in their study. Of 8 PBDE congeners tested in our ERα assay using CHO-K1 cells, BDEs 28, 47, and 100 were weakly estrogenic, whereas BDEs 15, 85, 99, 153, and 209 showed little activity (Figure 3A). These results agree well with those of the studies of Hamers et al. (2006) and Meerts et al. (2001). In addition, we demonstrated that three metabolites (4′-HO-BDE-17, 4′-MeO-BDE-17, and 4-HO-BDE-42) were also estrogenic, with 4′-HO-BDE-17, in particular, showing the most potent estrogenic activity among the 16 tested compounds (Figure 3A). Recently, Mercado-Feliciano and Bigsby (2008) also reported that 4′-HO-BDE-17 showed the most potent estrogenic activity among six mono-HO-PBDE derivatives from a commercial PBDE mixture (DE-71) tested in a trans-activation assay using the stable reporter system ER-positive BG1Luc4E2 ovarian cancer cells. Their results supported those from our study. On the other hand, to date, there is no information on the estrogenicity of MeO-PBDEs. In the present study, we found that 4′-MeO-BDE-17 has weak estrogenic activity via ERα, although its activity was approximately 15-fold lower than that of 4′-HO-BDE-17. This difference indicates that MeO-PBDEs are weaker ERα agonists than their corresponding HO-PBDEs, suggesting that the hydroxyl group might play an important role in the binding affinity to ERα. Furthermore, we found that BDE-99, BDE-153, 4′-HO-BDE-17, 4′-HO-BDE-49, 4′-MeO-BDE-49, and 4-MeO-BDE-90 showed dose-dependent ERα antagonistic activity in the presence of 1 × 10^−11^ M E_2_ (Figure 3B). Excluding BDE-153, which has been reported as an ER antagonist (Meerts et al. 2001), we newly identified that these five compounds have antiestrogenic properties via ERα. Regarding the structure–activity relationship of these compounds, the common structure of 2,2′,4,4′,5-substituted diphenyl ethers may be important in inhibiting ERα-mediated estrogenic activity, because BDE-99, 4′-HO-BDE-49, and 4′-MeO-BDE-49 showed similar ERα antagonistic activity. In addition, although 4′-HO-BDE-17 is a very potent ERα agonist, it also shows ERα antagonistic activity at a high dose (1 × 10^−5^ M), which explains the decrease in its agonistic activity shown in Figure 3A. However, 4-HO-BDE-90 did not induce or inhibit the ERα-mediated transcriptional activity, suggesting that presence of two bromine atoms next to the *para*-HO group may interfere with binding to ERα, or the absence of the bromine atoms next to the *para*-HO group (an isolated *para*-HO group) may be important for the induction of high estrogenic activity. Taken together, these results suggest that several PBDE congeners and their HO and MeO analogues might act as ERα agonists and/or antagonists, which is similar to the results for PCB observed previously by Connor et al. (1997) and Korach et al. (1988).

Although the results we obtained from the ERβ assay were similar to those of the ERα assay, with 4′-HO-BDE-17 showing the strongest estrogenic activity, we found that BDE-100 showed antagonistic activity in the ERβ assay despite showing agonistic activity in the ERα assay. This suggests that the bromine atom on the 6-position may play an important role on the discrepancy between ERα- and ERβ-mediated transcriptional activities. The overall structure of the ERβ-ligand binding domain is very similar to that of the ERα-ligand binding domain, and most of the compounds demonstrate similar binding affinities to and transcriptional activities with ERα and ERβ (Kuiper et al. 1997). However, some chemicals have been reported to have activities that vary according to ER subtype. The bisphenolic metabolite of methoxychlor, 2,2-bis-(*p*-hydroxyphenyl)-1,1,1-trichloroethane (HPTE), for example, has been shown to act as an ERα agonist and ERβ antagonist in human hepatoma (HepG2) cells (Gaido et al. 2000). We also found that several phthalates and HPTE act as an ERα agonist and ERβ antagonist in CHO-K1 cells (Takeuchi et al. 2005). The mechanism underlying this variation in activity via ERα and ERβ is thought to involve the helix 12 region present in both estrogenic receptors, and the agonist orientation of helix 12 in ERβ has been reported to be unstable and thus more likely to be antagonistic than that in ERα (Gaido et al. 2000). Thus, PBDEs may also be able to stabilize helix 12 in the agonist orientation for ERα but not for ERβ. As a result, we have provided the first evidence that BDE-100 has different transcriptional activities via ERα and ERβ.

*In vivo* and *in vitro* antiandrogenicity of PBDEs have been reported by Stoker et al. (2005) and Hamers et al. (2006). Their studies indicated that, among principle PBDE congeners, BDE-100 showed potent anti androgenic activity. In the present study, although none of the tested compounds showed any androgenic activity, 12 PBDEs, including metabolites, possessed AR antagonistic activity, with 4′-HO-BDE-17 showing the most potent antiandrogenic activity of all compounds tested, followed by BDE-100 (Figure 5). These results suggest that the PBDEs with three to five bromine substitutions have antiandrogenic activities via AR, and 4′-HO-BDE-17 acts as a potent AR antagonist in addition to being a potent ERα and ERβ agonist. On the other hand, 4-HO-BDE-90 had reduced binding ability to AR as well as to ERs compared with that of 4-MeO-BDE-90, possibly because of the presence of two bromines adjacent to the hydroxyl group on the phenyl group. Thus, many PBDEs, including their metabolites, simultaneously show both ERα/β-agonistic and ERα/β- and AR-antagonistic activities. We and other researchers have already shown that a large number of environmental estrogens are also anti androgenic (Kojima et al. 2004; Sohoni and Sumpter 1998; Vinggaard et al. 1999). The transcriptional activities of these active PBDEs via ERα/β and/or AR may require the number of bromine substitutions and their position on the diphenyl ether structure. In a previous study (Kojima et al. 2004) we demonstrated that the diphenyl ether-type herbicides chloronitrofen and chlomethoxyfen possess the most potent AR antagonistic activities (RIC_20_ = 4.3 × 10^−8^ M and 6.8 × 10^−8^ M, respectively) along with 4′-HO-BDE-17. These results suggest that halogenated compounds having a diphenyl ether structure may be potentially potent AR antagonists, and thereby antiandrogenic PBDEs of greater potency than 4′-HO-BDE-17 may exist among other PBDE isomers not tested in this study.

Glucocorticoids are steroid hormones essential for normal growth and development, for liver and immune functions, and for mediated stress responses. The actions of glucocorticoids are mediated by binding to the GR, a member of the nuclear receptor superfamily. To our knowledge, no PBDE congeners or their metabolites have previously been reported to possess GR agonistic or antagonistic activity. In the present study, we provided the first evidence that 7 of the 16 PBDE compounds tested have similar GR antagonistic activity at concentrations of 10^−6^ M order. Interestingly, these compounds include the penta-BDEs BDE-85, BDE-99, and BDE-100, as well as 4′-HO-BDE-49 and 4′-MeO-BDE-49, which correspond structurally to BDE-99. In a recent study, Molina-Molina et al. (2006) reported that M2 (3′,5′-dichloro-2-hydroxy-2-methyl-but-3-enanilide), one of the two primary metabolites of the dicarboximide fungicide vinclozolin, showed GR antagonistic activity. However, the structures common to non-steroidal GR antagonists might be not fully clarified because there have been few reports on chemicals with known GR activity. Thus, although the potencies of the PBDEs and their metabolites are low when compared with RU-486, a known steroidal GR antagonist, their potential for an additive or synergistic effect via GR should be taken into consideration in the risk assessment process.

Several studies have reported that PBDEs and HO-PBDEs bind competitively with human transthyretin (TTR), a transport protein for the thyroid hormones T_3_ and thyroxine (T_4_), thereby hampering the transportation of thyroid hormone (Meerts et al. 2000; Richardson et al. 2008; Zhou et al. 2001, 2002). It is of particular interest that the binding activity of 4-HO-BDE-42 and 4′-HO-BDE-49 for TTR is 1,400-fold higher than that of their parent BDE-47 and 3-fold higher than that of natural ligand T_4_ (Hamers et al. 2008). On the other hand, few studies have addressed the binding ability of PBDEs with TR, except for a competitive binding study using rat TR by Kitamura et al. (2008). In the present study, we investigated hTRα_1_/β_1_ agonistic and antagonistic activity of 16 PBDEs and metabolites using CHO-K1 cell–based reporter gene assays. As a result, we demonstrated for the first time that only 4-HO-BDE-90 has antagonistic activity against both TRα_1_ and TRβ_1_, which is similar to the results for TBBPA (Kitamura et al. 2005b). This result supports those from Kitamura et al. (2008), who reported that 4-HO-BDE-90 bound to rat TR. The TR antagonists TBBPA and 4-HO-BDE-90 have common structures, including a 4-hydroxyl group and two bromine substitutions adjacent to the hydroxyl group on the phenyl group, that are also shared by HO-PCBs as reported in some studies (Cheek et al. 1999; Kitamura et al. 2005a). This finding may indicate the essential structural factors for binding to TR, although binding affinities of these compounds might be much lower than those of the endogenous ligands T_3_ and T_4_.

As discussed above, our study suggests that principle PBDE congeners found in human tissues and their HO and MeO metabolites have multiple endocrine-disrupting effects via nuclear hormone receptors. In particular, the *para*-HO-PBDEs used in this study have been reported to be HO metabolites found in blood samples from rats and mice administrated seven equimolar PBDE congeners (BDEs 47, 99, 100, 153, 154, 183, and 200) and a commercial penta-PBDE mixture DE-71 containing BDEs 47, 99, 153, and 154, respectively (Malmberg et al. 2005; Qui et al. 2007). Therefore, it has been considered that these metabolites may in fact act as endocrine disruptors in humans as well as in rodents. Most recently, two reports on PBDE metabolites identified in human blood samples have been published. Athanasiadou et al. (2008) reported that, of several HO-PBDEs, 6-HO-BDE-47, 4′-HO-BDE-17, and 4′-HO-BDE-49 were the dominant phenolic metabolites in blood samples from children living or working at a municipal waste disposal site in Managua, Nicaragua. On the other hand, Qui et al. (2009) reported that the metabolite profile in blood samples from pregnant women and their newborn babies living in the United States was very different from the results of Athanasiadou et al. (2008): Two metabolites formed without a bromine shift (5-HO-BDE-47 and 5′-HO-BDE-99) were more abundant, although 4′-HO-BDE-49 and 4-HO-BDE-42 were detected at much lower concentrations in some of the 20 human blood samples. This also suggests that there are differences in the metabolic profile of human individuals and between humans and rodents because of species differences in CYP enzyme expression. Thus, further analytical study on PBDE metabolites, including MeO derivatives, in humans is required for health risk assessment.

Transactivation assays using CHO-K1 cells revealed that PBDEs and their metabolites act as an agonist and/or antagonist via ERα/β, AR, GR, and TRα/β, suggesting that they might have the potential to affect the endocrine system. The ligand-dependent activation of nuclear receptors requires ligand-dependent association of protein cofactors and basal transcription factors, for whose expression levels differ from cell to cell (McKenna et al. 1999). Furthermore, there is some discrepancy in the cellular metabolic ability against chemicals between CHO-K1 and other cell lines such as hepatocarcinoma cells. Thus, there is a possibility that use of different cells yields different results. However, we think that it is important to evaluate several receptor activities of chemicals under the same cell conditions using one highly sensitive and specific assay method, and the reporter gene assays using CHO-K1 cells are useful for identifying endocrine disruptors.

Interestingly, the antiestrogenic and anti-androgenic properties of PBDE-153 found in this study are very similar to those of PCB-153, which is known to accumulate in human blood (Bonefeld-Jørgensen et al. 2001). These effects against ER and AR may reflect structural resemblance between PBDE and PCB. Thus, because many PBDEs, including those not used in this study, are speculated to have nuclear hormone receptor activities, we plan to study their effects, particularly those of accumulative PBDE metabolites such as 6-HO-BDE-47, 5-HO-BDE-47, and 5′-HO-BDE-99 recently found in human blood (Athanasiadou et al. 2008; Qui et al. 2009). In addition, another study has indicated that PBDEs are able to induce the expression of CYP3A11- and CYP2B10-metabolizing enzymes by functioning as a ligand of pregnane X receptor (PXR), a member of the nuclear receptor superfamily (Pacyniak et al. 2007). These enzymes contribute to the metabolization of not only xenobiotics, such as drugs, but also endogenous hormones. Therefore, PBDEs may also indirectly disrupt the endocrine system by interacting with nuclear receptors other than hormone receptors, subsequently altering metabolizing enzyme activity. Further study is required to understand the structure–activity relationship between PBDEs and their metabolites and nuclear hormone receptors, and their pleiotropic effects against other nuclear receptors such as PXR and constitutive androstane receptor.

## Figures and Tables

**Table 1:** Comparison of agonistic and antagonistic activities of PBDEs and their HO and MeO metabolites against ERα, ERβ, AR, GR, TRα_1_, and TRβ_1_.

	Agonistic activity: REC_20_^a^ (M)		Antagonistic activity: RIC_20_^b^ (M)					
Compounds	ERα	ERβ	ERα	ERβ	AR	GR	TRα_1_	TRβ_1_
E_2_	2.5 × 10^−12^	5.3 × 10^−12^	—	—	—	—	—	—

TAM	—	—	6.0 × 10^−9^	7.0 × 10^−9^	—	—	—	—

HF	—	—	—	—	1.8 × 10^−8^	—	—	—

RU-486	—	—	—	—	—	5.7 × 10^−9^	—	—

TBBPA	—	—	—	—	—	—	4.1 × 10^−6^	9.6 × 10^−6^

BDE-15	NE	NE	NE	NE	NE	NE	NE	NE

BDE-28	6.7 × 10^−6^	NE	NE	NE	1.3 × 10^−6^	NE	NE	NE

BDE-47	5.0 × 10^−6^	NE	NE	NE	8.1 × 10^−7^	NE	NE	NE

BDE-85	NE	NE	NE	NE	1.1 × 10^−6^	7.0 × 10^−6^	NE	NE

BDE-99	NE	NE	5.7 × 10^−6^	9.2 × 10^−6^	1.4 × 10^−6^	7.7 × 10^−6^	NE	NE

BDE-100	4.0 × 10^−6^	NE	NE	4.3 × 10^−6^	2.1 × 10^−7^	8.5 × 10^−6^	NE	NE

BDE-153	NE	NE	3.3 × 10^−6^	4.4 × 10^−6^	NE	NE	NE	NE

BDE-209	NE	NE	NE	NE	NE	NE	NE	NE

4′-HO-BDE-17	1.9 × 10^−7^	2.1 × 10^−7^	8.8 × 10^−6^	NE	8.6 × 10^−8^	6.7 × 10^−6^	NE	NE

4′-MeO-BDE-17	2.5 × 10^−6^	NE	NE	NE	8.2 × 10^−7^	NE	NE	NE

4-HO-BDE-42	2.9 × 10^−6^	3.6 × 10^−6^	NE	NE	1.1 × 10^−6^	NE	NE	NE

4-MeO-BDE-42	NE	NE	NE	NE	1.7 × 10^−6^	NE	NE	NE

4′-HO-BDE-49	NE	NE	2.3 × 10^−6^	3.6 × 10^−6^	1.5 × 10^−6^	5.5 × 10^−6^	NE	NE

4′-MeO-BDE-49	NE	NE	4.4 × 10^−6^	6.1 × 10^−6^	8.5 × 10^−7^	6.2 × 10^−6^	NE	NE

4-HO-BDE-90	NE	NE	NE	NE	NE	NE	8.1 × 10^−6^	7.3 × 10^−6^

4-MeO-BDE-90	NE	NE	2.6 × 10^−6^	7.6 × 10^−6^	2.5 × 10^−6^	2.5 × 10^−6^	NE	NE

NE, no effect (REC_20_ or RIC_20_ > 1 × 10^−5^ M).

a Concentration of the test compound showing 20% of the agonistic activity of 10^−9^ M E_2_.

b Concentration of the test compound showing 20% of the antagonistic activity of 1 × 10–11 M E_2_ via ERα, 1 × 10^−10^ M E_2_ via ERβ, 1 × 10^−10^ M DHT via AR, 3 × 10^−8^ M via GR, 1 × 10^−8^ M via TRα, or 1 × 10^−8^ M via TRβ.
